# Combined high-intensity interval and resistance training improves cardiorespiratory fitness more than high-intensity interval training in young women with overweight/obesity: a randomized controlled trial

**DOI:** 10.3389/fendo.2024.1450944

**Published:** 2024-11-11

**Authors:** Yifei Wang, Xin Yang, Jiamei Deng, Zhenshan Wang, Dongxue Yang, Yanbai Han, Hongli Wang

**Affiliations:** College of Physical Education and Health, Guangxi Normal University, Guilin, Guangxi, China

**Keywords:** high-intensity interval training, resistance training, cardiorespiratory fitness, obesity, randomized controlled trial

## Abstract

**Objective:**

This study aimed to compare the effects of high-intensity interval training (HIIT) combined with resistance training (RT) versus HIIT alone on body composition, cardiorespiratory fitness and glycolipid metabolism in young women with overweight/obesity.

**Methods:**

This randomized controlled trial divided 40 subjects into an experimental group (HIIT+RT) and a control group (HIIT). Both groups underwent training three times per week for eight weeks. Body composition, cardiorespiratory fitness and glycolipid levels were assessed before and after the intervention.

**Results:**

The results revealed that compared to baseline, both the experimental and control groups showed significant improvements in body weight, body mass index (BMI), Body fat, waist circumference (WC), waist-hip ratio (WHR), peak oxygen uptake (VO_2_peak), vital capacity (VC), oxygen pulse (VO_2_/HR), minute ventilation, resting heart rate, blood oxygen saturation, blood pressure, fasting blood glucose, triglycerides and high-density lipoprotein cholesterol (*p*<0.05). Additionally, a significant increase in muscle mass and a significant reduction in 2-hour postprandial glucose were observed in the experimental group (*p*<0.05). Muscle mass (mean difference: 2.75%), VO_2_peak (mean difference: 1.61 mL/min/kg), VC (mean difference: 334mL), and VO_2_/HR (mean difference: 0.51mL/beat) showed greater improvement in the HIIT+RT group compared to the HIIT group (*p*<0.05).

**Conclusion:**

In conclusion, an 8-week regimen of either combined HIIT and RT or HIIT significantly improves body composition, cardiorespiratory fitness and glycolipid metabolism in women with overweight/obesity. However, the combined training appears to offer more benefits than HIIT alone. Further research is needed to evaluate the long-term effects and feasibility of combined training.

**Trial registration:**

https://www.chictr.org.cn/, identifier ChiCTR2300075961.

## 1 Introduction

The increasing global incidence of overweight and obesity, particularly in younger populations, heightens concerns regarding health risks (1, 2). The Body Mass Index (BMI) and Waist Circumference (WC) are frequently employed as standard measures to identify cases of overweight and obesity. Elevated BMI and increased WC during adulthood are crucial risk factors for cardiovascular diseases, hypertension, diabetes, musculoskeletal disorders, cancer, as well as morbidity and mortality (3). Compared to individuals with normal weight, patients with obesity often have poorer cardiopulmonary function (4), which can lead to hypoxia and breathing difficulties, resulting in cardiopulmonary failure (5). Additionally, due to fat accumulation, patients with obesity have lower insulin sensitivity, making them prone to hyperglycemia and lipid abnormalities, resulting in metabolic disorders and increasing the risk of chronic diseases like cardiovascular conditions (6). Nowadays, obesity has become a strong predictor of higher all-cause mortality rates, which cannot be overlooked, especially in young people (7). Therefore, weight reduction, enhancement of cardiopulmonary function, and improvement of glycolipid metabolism are particularly crucial for individuals with overweight and obesity.

Regular physical exercise is one of the most effective strategies for preventing and treating overweight and obesity, which improves overall health and reduces complications related to obesity (8). High-intensity interval training (HIIT), distinguished by brief, recurrent periods of vigorous aerobic activity interspaced with intervals of low-intensity rest or recovery, is a recommended and efficient exercise modality for treating obesity (9). A meta-analysis has shown that, compared to moderate-intensity continuous training, HIIT is more beneficial in improving the cardiopulmonary health in patients with obesity and enhances exercise efficiency (10, 11). A higher level of cardiopulmonary function can also mitigate the adverse effects of obesity on cardiovascular health (12), thereby reducing the incidence and mortality risks of cardiovascular diseases (13). Additionally, another study confirmed that HIIT can improve body composition and metabolic abnormalities in women with obesity, with better compliance (14). Tabata training acknowledged as one of the most effective forms of HIIT (including 20 seconds of high-intensity exercise followed by 10 seconds of rest), induces various positive exercise adaptations in healthy individuals (15), but its benefits for patients with obesity are less understood. Resistance Training (RT) is recognized by the American College of Sports Medicine as a viable exercise modality, widely applied in treating obesity and positively impacting metabolic health (16, 17). After the HIIT and RT interventions, men and women also responded to certain gender differences, such as more active heart rate variability, lower body fat percentage, and more pronounced strength bonuses (18–20). To date, no studies have conclusively shown whether a combination of these two exercise modalities produces synergistic positive or negative interference effects in patients with obesity, and whether combined training is more effective than HIIT alone in treating obesity remains unknown.

Therefore, this study aims to compare the effects of HIIT based on the Tabata protocol combined with RT versus HIIT alone on the body composition, cardiorespiratory fitness and glycolipid metabolism in young women with overweight and obesity.

## 2 Materials and methods

### 2.1 Design

This is an 8-week, single-center, parallel-group, two-arm randomized controlled trial (chictr.org.cn: identifier: ChiCTR2300075961) employing concealed allocation and blinded assessment. Participants were randomly assigned in a 1:1 ratio to the experimental intervention group (HIIT+RT) and the control intervention group (HIIT). The study was conducted in the Exercise Science Laboratory and gym at the College of Physical Education and Health, Guangxi Normal University. We recruited 40 voluntary and eligible subjects with overweight or obesity, using opaque sealed envelopes containing computer-generated random sequences to achieve concealed allocation, a task completed by an external assistant not involved in the trial. The percentage of overweight and obesity was 47% and 53% in the HIIT+RT group and 37% and 63% in the HIIT group, respectively.

All baseline and post-intervention measurements were conducted by the same professional, who was unaware of the study protocol and therefore blind to group assignments. All participants were instructed not to smoke, drink alcohol, or engage in vigorous exercise 48 hours before the measurements. Given the characteristics of the exercise regimen, it was impractical to blind participants and trainers to the nature of the training.

### 2.2 Participants

We recruited subjects through online social media and offline promotions. The inclusion criteria were: female, aged 18-25 years, with a BMI ≥ 24 kg/m² (21), irregular physical exercise habits, and willingness to participate in the experiment. Exclusion criteria included current use of antihypertensive or lipid-lowering medications; weight change greater than 4 kg in the three months prior to the study (22); and any pulmonary, cardiovascular, muscular, neurological, or other diseases unsuitable for exercise intervention. This study protocol was approved by the Human Ethics Committee of Guangxi Normal University (20230913001). Furthermore, all procedures adhered to the guidelines of the *Declaration of Helsinki*. Prior to enrollment, each participant provided their informed consent by signing a consent form.

### 2.3 Intervention

All subjects were assigned to a 50-minute exercise session three times per week for 8 weeks.

#### 2.3.1 Experimental group

Each workout session for the experimental group began with a 10-minute warm-up (60%-70% of maximum heart rate [HRmax]), followed by 20 minutes of Tabata training (4 sets of 4 minutes with a 1-minute rest between sets), 15 minutes of resistance training, and ending with a 5-minute stretching and relaxation. The Tabata training protocol integrates dynamic movements including jumping jack, crotch clap, knee-to elbow, squat, side knee raise, kick back, touch foot and high knees, etc. The resistance training was equipment-based, and we designed a three-part training program suitable for obese patients, following the ACSM guidelines (23). The muscles targeted included the biceps brachii, triceps brachii, trapezius, deltoids, pectorals, latissimus dorsi, gluteus, quadriceps, hamstrings, and calf muscles. For the training intensity, the maximum weight an individual could lift in one repetition (1RM) was used. The object of the workout was to complete 3 sets of 10 repetitions of each movement at 70% 1RM (24). The 1RM test was repeated weekly. In their training notes, participants detailed the weight, repetitions, and sets of each exercise. Tabata and resistance training specific regimens are detailed in (**Supplementary Table S1, S2**).

#### 2.3.2 Control group

Each session for the control group involved only high-intensity interval training, starting with a 10-minute warm-up, followed by 35 minutes of Tabata training (7 sets of 4 minutes with a 1-minute rest between sets), and ending with a 5-minute cooldown. The Tabata training content was consistent with that of the experimental group.

### 2.4 Procedures

Due to the high intensity of the exercise, all participants underwent a week of adaptive training (including familiarization with and use of resistance equipment) before the official training. They were then scheduled for three sessions per week for eight weeks (2). We required all participants to abstain from other types of exercise during the intervention period, except for the designated regimen. Each participant exercised under the strict supervision of a professional fitness coach. HIIT followed the Tabata protocol-based training method (20 seconds of high-intensity exercise followed by 10 seconds of rest) (15). Participants were encouraged to repeat the action as many times as possible during the 20-second exercise phase, followed by a 10-second rest. Keep the Tabata training intensity at 85%-90% HRmax for the first four weeks and increase it to 90%-95% HRmax for the second four weeks. Heart rate monitors (Polar H9, Finland) were used to adjust the exercise intensity to achieve the desired level while ensuring safety (25). Additionally, we did not specifically restrict the participants’ diets, asking them to maintain their habitual diets. A continuous three-day dietary survey (including two weekdays and one weekend day) was conducted before and after the intervention for all participants (14), using the Boohee software (Shanghai, China) for analysis. This determined the participants’ dietary composition and daily energy intake. They were instructed to fill out a 24-hour dietary recall questionnaire under the guidance of researchers.

### 2.5 Outcome measures

#### 2.5.1 Body composition

The subjects’ height, body weight, BMI, body fat, and muscle mass were assessed using a bioelectrical impedance analyzer (GAIA KIKO, Korea). Prior to the measurements, participants were instructed to remove their footwear, socks, headwear, and heavy outer garments. They were also prohibited from carrying any objects. During the measurement process, participants held onto the handle and positioned their fingers in the standard position with their upper limbs extended at a 30° angle. WC was measured using a tape measure and waist-hip ratio (WHR) was calculated using a specific formula.

#### 2.5.2 Cardiorespiratory fitness

Peak oxygen uptake (VO_2_peak), minute ventilation (VE), respiratory quotient (RQ), oxygen pulse (VO_2_/HR) and HRmax were measured using a portable gas metabolism analyzer (COSMED K5, Italy). VO_2_peak refers to the maximum value of oxygen content that can be ingested by the body during maximum intensity exercise, representing the limit level of the body’s ability to supply oxygen. Subjects performed a graded exercise test on a cycle ergometer, starting from a warm-up at 0 Watts, with a 20W increment every two minutes, maintaining 60 rpm. The assessment concluded when the participant reached exhaustion, oxygen consumption reached its peak, and the respiratory exchange ratio surpassed 1.10 (22). The Rating of Perceived Exertion was asked at each increment. HRmax was defined as the highest heart rate recorded during the test.

Resting heart rate (RHR) and blood pressure (BP) were measured using a heart rate monitor (Polar H9, Finland) and an electronic BP monitor (OMRON, HEM-7136, Japan), respectively. Participants were allowed ample rest before measurement, and these were completed before conducting other tests. Vital capacity (VC) was measured using an electronic spirometer (Wanqing, China), with three measurements taken per person and the average value calculated. Blood oxygen saturation (SpO_2_) was tested using a finger clip pulse oximeter (medisana, Germany).

#### 2.5.3 Glycolipid metabolism

To avoid the influence of biological rhythms on test results, blood samples were collected after fasting for 12 hours, between 08:00 and 09:00 AM, at the Yanshan District People’s Hospital by professional medical staff. The samples were then transported to the laboratory for standardized analysis. Fasting blood glucose (FBG) was determined using the hexokinase method. Enzymatic methods were employed to measure total cholesterol (TC), triglycerides (TG), high-density lipoprotein cholesterol (HDL-C), and low-density lipoprotein cholesterol (LDL-C). 2-hour postprandial glucose (2hPG) levels were measured using a glucometer (yuwell660, China) to take the average of three meals a day from the finger-tip venous blood.

### 2.6 Sample size

The sample size for the randomized controlled trial (comparison of means between two groups) was calculated. The VO_2_peak measurement was chosen as the outcome indicator. A two-sided test was set, with equal sample sizes, α=0.05, and a test power of 1- β=80%. Based on previous studies, the mean VO_2_peak levels for the experimental and control groups were 29.0 (mL/min/kg) and 26.4 (mL/min/kg), respectively, with a standard deviation of 2.0 (mL/min/kg) (26). Using PASS 15 software, it was calculated that a minimum sample size of 11 individuals per group was required. After factoring in a 20% dropout rate, a minimum of 14 subjects per group was deemed necessary.

### 2.7 Statistical analyses

Data analyses were performed in IBM SPSS Statistics 27 and data were represented as mean and standard deviation (SD). We used paired t-tests to compare post- and pre-intervention means within groups. Analysis of covariance (ANCOVA) was conducted to evaluate the differences in means between groups for each variable both prior to and following the intervention, with the pre-assessment data used as covariates. The effect sizes (ES) of the differences between groups were quantified using a partial eta squared, with thresholds for categorizing effect sizes as small, medium, and large were set at 0.01, 0.06, and 0.14, respectively. Within-group differences were computed and displayed employing Cohen’s d values, the thresholds for effect size magnitude were defined as: ES < 0.2 (trivial), 0.2 ≤ ES < 0.5 (small), 0.5 ≤ ES < 0.8 (medium), ES ≥ 0.8 (large) (27). The significance level was set at *p* < 0.05.

## 3 Results

### 3.1 Study population

**Figure 1:**
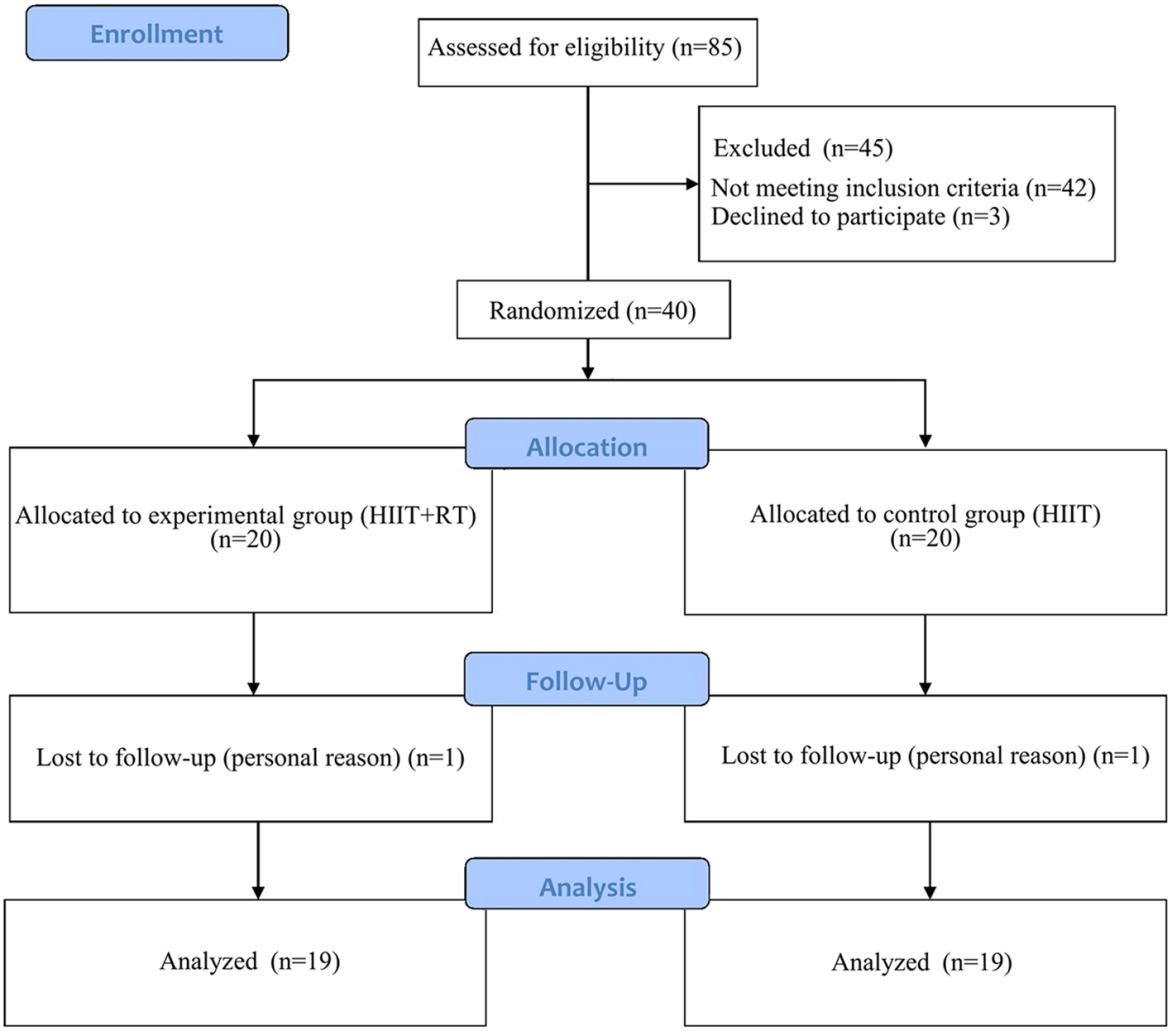
CONSORT flow diagram.

A total of 85 patients with overweight and obesity were assessed for eligibility, and after excluding 45 patients (including those not meeting the inclusion criteria n=42, and refusal to participate n=3), 40 subjects participated in the study (**Figure 1**). Two subjects withdrew during the follow-up period for personal reasons, while the remaining 38 subjects successfully completed the 8-week training program by attending three sessions per week. The data from these 38 subjects were included in the final analyses. A researcher recorded weekly attendance. At the outset, the Baseline characteristics of the two groups showed no significant disparities (**Table 1**).

**Table 1:** Baseline characteristics.

	HIIT+RT (n=19)	HIIT (n=19)	*p*-value
Age(year)	22 (1.8)	22 (2.2)	0.94
Height (cm)	160.9 (6.8)	159.7 (6.0)	0.55
Weight (kg)	74.5 (9.7)	73.1 (9.9)	0.67
BMI (kg/m^2^)	28.7 (2.7)	28.6 (3.0)	0.95
Body fat (%) Muscle mass (%) WC (cm) Waist-hip ratio SBP (mmHg)	34.71 ± 3.09 44.20 ± 4.36 86.24 ± 5.32 0.85 ± 0.03 121.6 (10.0)	34.21 ± 2.75 43.68 ± 4.13 86.61 ± 6.40 0.85 ± 0.03 118.1 (6.7)	0.51 0.72 0.89 0.41 0.21
DBP (mmHg)	84.2 (9.3)	81.3 (7.3)	0.29
SpO_2_ (%)	96 (1.0)	96 (1.3)	0.89
RHR (bpm)	74.9 (5.0)	73.0 (4.9)	0.23
HRmax (bpm)	185.8 (4.9)	184.0 (5.7)	0.29
Vital capacity (mL)	3854 (684)	3739 (639)	0.60
VO_2_peak (mL/min/kg)	23.9 (3.7)	22.3 (3.2)	0.17
Respiratory quotient VO_2_/HR (mL/beat) VE (L/min)	1.20 (0.05) 9.80 (0.98) 48.7 (10.4)	1.21 (0.06) 9.34 (1.00) 45.9 (9.5)	0.50 0.16 0.39
FBG (mmol/L)	4.86 (0.34)	4.96 (0.29)	0.34
2hPG (mmol/L)	6.35 (0.96)	6.23 (0.71)	0.65
TG (mmol/L)	1.30 (0.39)	1.34 (0.48)	0.76
TC (mmol/L)	4.78 (0.48)	4.82 (0.78)	0.84
HDL-C (mmol/L)	1.39 (0.21)	1.44 (0.28)	0.54
LDL-C (mmol/L)	2.51(0.45)	2.60 (0.46)	0.54
Energy intake (Kcal/day)	3016 (211)	3100 (181)	0.20

Data are expressed as the mean (standard deviation).

BMI, body mass index; WC, Waist circumference; DBP, diastolic blood pressure; FBG, fasting blood glucose; 2hPG, 2-hour postprandial glucose; HRmax, maximum heart rate; HDL-C, high-density lipoprotein cholesterol; LDL-C, low-density lipoprotein cholesterol; RHR, resting heart rate; SBP, systolic blood pressure; SpO_2_, Blood oxygen saturation; TG, triglyceride; TC, total cholesterol; VO_2_peak, peak oxygen uptake; VO_2_/HR, oxygen pulse; VE, minute ventilation.

### 3.2 Body composition

Compared to the pre-intervention period, the HIIT+RT group exhibited significant reductions in body weight (mean difference: -1.66 kg), BMI (mean difference: -0.65 kg/m^2^), body fat (mean difference: -1.47%), WC (mean difference: -1.21 cm), and WHR (mean difference: -0.02) while demonstrating an increase in muscle mass (mean difference: 1.82%) (p<0.05). The HIIT group also showed significant decreases in body weight (mean difference: -1.49 kg), BMI (mean difference: -0.68 kg/m^2^), body fat (mean difference: -0.70%), WC (mean difference: -1.37 cm), and WHR (mean difference: -0.02) (p<0.05). In comparison to the HIIT group alone, combined training significantly improved muscle mass (mean difference: 2.75%) among subjects (p<0.05), with no significant differences observed in terms of body weight, BMI, body fat, WC and WHR between the two groups (p>0.05) (**Table 2**).

**Table 2:** Changes in indicators of body composition in the HIIT+RT and HIIT groups.

Measurement	Group	PRE	POST	Within-group differences	Between-group differences	Effect size
		Mean (SD)	Mean (SD)	Mean (95%CI)	Mean (95% CI)	
Body weight (kg)						
	HIIT+RT	74.48 ± 9.75	72.82 ± 9.67	-1.66 (-2.68 to -0.65) ^**^	NA	0.791
	HIIT	73.11 ± 9.85	71.62 ± 9.44	-1.49 (-2.20 to -0.78) ^**^	NA	1.006
	HIIT+RT vs HIIT	NA	NA	NA	-0.12 (-1.30 to 1.07)	0.001
BMI (kg/m^2^)						
	HIIT+RT	28.67 ± 2.66	28.03 ± 2.66	-0.65 (-1.05 to -0.25) ^**^	NA	0.778
	HIIT	28.62 ± 3.00	27.94 ± 2.90	-0.68 (-1.01 to -0.35) ^**^	NA	0.998
	HIIT+RT vs HIIT	NA	NA	NA	0.04 (-0.46 to 0.53)	0.001
Body fat (%)						
	HIIT+RT	34.71 ± 3.09	33.24 ± 3.27	-1.47 (-2.04 to -0.90) ^**^	NA	1.245
	HIIT	34.21 ± 2.75	33.51 ± 2.82	-0.70 (-1.18 to -0.22) ^**^	NA	0.706
	HIIT+RT vs HIIT	NA	NA	NA	-0.76 (-1.49 to -0.03)	0.113
Muscle mass (%)						
	HIIT+RT	44.20 ± 4.36	46.02 ± 4.29	1.82 (1.20 to 2.43) ^**^	NA	1.431
	HIIT	43.68 ± 4.13	42.78 ± 4.29	-0.90 (-1.82 to 0.02)	NA	0.472
	HIIT+RT vs HIIT	NA	NA	NA	2.75 (1.68 to 3.82) ^**^	0.437
WC (cm)						
	HIIT+RT	86.24 ± 5.32	85.03 ± 4.86	-1.21 (-1.96 to -0.46) ^**^	NA	0.782
	HIIT	86.61 ± 6.40	85.24 ± 6.74	-1.37 (-2.09 to -0.65) ^**^	NA	0.913
	HIIT+RT vs HIIT	NA	NA	NA	0.15 (-0.86 to 1.15)	0.002
Waist-hip ratio						
	HIIT+RT	0.85 ± 0.03	0.83 ± 0.03	-0.02 (-0.03 to -0.01) ^**^	NA	0.926
	HIIT	0.85 ± 0.03	0.83 ± 0.04	-0.02 (-0.02 to -0.01) ^**^	NA	0.995
	HIIT+RT vs HIIT	NA	NA	NA	-0.002 (-0.014 to 0.009)	0.004

BMI, Body mass index; WC, Waist circumference; ^*^: *p*<0.05; ^**^: *p*<0.01.

### 3.3 Cardiorespiratory fitness

In comparison to initial measurements, significant improvements in VO_2_peak (mean differences: 3.41 mL/min/kg and 2.24 mL/min/kg), VE (mean differences:16.26 L/min and 15.59 L/min), VO_2_/HR (mean differences:1.54mL/beat and 1.06mL/beat), VC (mean differences:578 mL and 278 mL), RHR (mean differences: -1.79 bpm and -2.68 bpm), SpO_2_ (mean differences: 1.21% and 1.16%), systolic BP (mean differences: -4.74 mmHg and -4.26 mmHg), and diastolic BP (mean differences:-3.47 mmHg and -3.11 mmHg) were observed in both the experimental and control groups post-intervention (*p*<0.05).

The experimental group was more beneficial in enhancements VO_2_peak (mean difference: 1.61 mL/min/kg), VO_2_/HR (mean difference: 0.51mL/beat), and VC (mean difference: 334mL) compared with the control group (*p*<0.05). However, there were no significant differences between the two groups in VE, RQ, RHR, HRmax, SpO_2_, systolic BP, and diastolic BP (*p*>0.05). Specific effect sizes are shown in (**Table 3**).

**Table 3:** Changes in indicators of cardiorespiratory fitness in the HIIT+RT and HIIT groups.

Measurement	Group	PRE	POST	Within-group differences	Between-group differences	Effect size
		Mean (SD)	Mean (SD)	Mean (95%CI)	Mean (95% CI)	
VO_2_peak(mL/min/kg)						
	HIIT+RT	23.89 (3.75)	27.30 (3.22)	3.41 (2.64 to 4.18) ^**^	NA	2.142
	HIIT	22.31 (3.18)	24.54 (3.26)	2.24 (0.90 to 3.58) ^**^	NA	0.805
	HIIT+RT vs HIIT	NA	NA	NA	1.61 (0.21 to 3.02) ^*^	0.134
VE (L/min)						
	HIIT+RT	48.69 (10.37)	64.95 (14.75)	16.26 (10.30 to 22.23) ^**^	NA	1.314
	HIIT	45.91 (9.51)	61.50 (11.80)	15.59 (9.00 to 22.19) ^**^	NA	1.139
	HIIT+RT vs HIIT	NA	NA	NA	1.95 (-6.30 to 10.20)	0.007
Respiratory quotient						
	HIIT+RT HIIT HIIT+RT vs HIIT	1.20 (0.05) 1.21 (0.06) NA	1.23 (0.08) 1.24 (0.10) NA	0.03 (-0.01 to 0.08) 0.03 (-0.01 to 0.06) NA	NA NA 0.01 (-0.05 to 0.06)	0.352 0.408 0.002
VO_2_/HR (mL/beat)						
	HIIT+RT HIIT	9.80 (0.98) 9.34 (1.00)	11.34 (1.13) 10.40 (1.19)	1.54 (1.21 to 1.87) ^**^ 1.06 (0.72 to 1.39) ^**^	NA NA	2.250 1.521
	HIIT+RT vs HIIT	NA	NA	NA	0.51 (0.03 to 0.98) ^*^	0.119
Vital capacity (mL)						
	HIIT+RT	3854 (684)	4431 (663)	578 (359 to 797) ^**^	NA	1.273
	HIIT	3739 (639)	4017 (509)	278 (103 to 452) ^**^	NA	0.768
	HIIT+RT vs HIIT	NA	NA	NA	334 (91 to 576) ^**^	0.182
RHR (bpm)						
	HIIT+RT	74.89 (4.98)	73.11 (6.09)	-1.79 (-2.80 to -0.78) ^**^	NA	0.853
	HIIT	72.95 (4.89)	70.26 (4.91)	-2.68 (-4.02 to -1.35) ^**^	NA	0.969
	HIIT+RT vs HIIT	NA	NA	NA	0.89 (-0.79 to 2.56)	0.032
HRmax (bpm)						
	HIIT+RT	185.84 (4.89)	187.05 (5.23)	1.21 (-0.21 to 2.63)	NA	0.412
	HIIT	184.00 (5.68)	185.42 (6.98)	1.42 (-0.33 to 3.18)	NA	0.390
	HIIT+RT vs HIIT	NA	NA	NA	-0.18 (-2.42 to 2.07)	0.001
SpO_2_ (%)						
	HIIT+RT	95.95 (1.03)	97.16 (0.96)	1.21 (0.58 to 1.85) ^**^	NA	0.920
	HIIT	95.89 (1.29)	97.05 (0.97)	1.16 (0.64 to 1.67) ^**^	NA	1.084
	HIIT+RT vs HIIT	NA	NA	NA	0.09 (-0.51 to 0.69)	0.003
SBP (mmHg)						
	HIIT+RT	121.58 (10.00)	116.84 (9.50)	-4.74 (-7.39 to -2.08) ^**^	NA	0.860
	HIIT	118.05 (6.74)	113.79 (7.98)	-4.26 (-8.25 to -0.28) ^*^	NA	0.516
	HIIT+RT vs HIIT	NA	NA	NA	0.62 (-3.82 to 5.06)	0.002
DBP (mmHg)						
	HIIT+RT	84.16 (9.31)	80.68 (9.22)	-3.47 (-5.44 to -1.50) ^**^	NA	0.850
	HIIT	81.26 (7.33)	78.16 (5.97)	-3.11 (-5.88 to -0.33) ^*^	NA	0.540
	HIIT+RT vs HIIT	NA	NA	NA	0.35 (-2.73 to 3.43)	0.002

DBP, diastolic blood pressure; HRmax, maximum heart rate; RHR, resting heart rate; SpO_2_, Blood oxygen saturation; SBP, systolic blood pressure; VO_2_peak, peak oxygen uptake; VE, minute ventilation; VO_2_/HR, oxygen pulse; ^*^, *p*<0.05; ^**^, *p*<0.01.

### 3.4 Glycolipid metabolism and energy intake

Compared to pre-intervention, the combined training significantly reduced participants’ FBG (mean difference: -0.42 mmol/L), 2hPG (mean difference: -0.60 mmol/L) and TG (mean difference: -0.16 mmol/L), while significantly increasing HDL-C (mean difference: 0.23 mmol/L) (*p*<0.05). In contrast, the HIIT group only improved FBG (mean difference: -0.38 mmol/L), TG (mean difference: -0.19 mmol/L), and HDL-C (mean difference: 0.16 mmol/L) (*p*<0.05).

The HIIT+RT group showed improvements in FBG, 2hPG, TG, TC, HDL-C, and LDL-C compared with the HIIT group, however, these variations did not reach a level of statistical significance (*p*>0.05). There was no significant change in energy intake in either the experimental or control groups (*p*>0.05) (**Table 4**).

**Table 4:** Changes in indicators of glycolipid metabolism and energy intake in the HIIT+RT and HIIT groups.

Measurement	Group	PRE	POST	Within-group differences	Between-group differences	Effect size
		Mean (SD)	Mean (SD)	Mean (95%CI)	Mean (95% CI)	
FBG (mmol/L)						
	HIIT+RT	4.86 (0.34)	4.44 (0.44)	-0.42 (-0.61 to -0.23) ^**^	NA	1.065
	HIIT	4.96 (0.29)	4.58 (0.45)	-0.38 (-0.64 to -0.12) ^**^	NA	0.695
	HIIT+RT vs HIIT	NA	NA	NA	-0.11(-0.40 to 0.19)	0.015
2hPG (mmol/L)						
	HIIT+RT	6.35 (0.96)	5.75 (0.78)	-0.60 (-1.08 to -0.12) ^*^	NA	0.604
	HIIT	6.23 (0.71)	5.97 (0.60)	-0.25 (-0.53 to 0.03)	NA	0.434
	HIIT+RT vs HIIT	NA	NA	NA	-0.27 (-0.68 to 0.15)	0.047
TG (mmol/L)						
	HIIT+RT	1.30 (0.39)	1.14 (0.29)	-0.16 (-0.30 to -0.03) ^*^	NA	0.571
	HIIT	1.34 (0.48)	1.16 (0.30)	-0.19 (-0.32 to -0.06) ^**^	NA	0.685
	HIIT+RT vs HIIT	NA	NA	NA	-0.01 (-0.12 to 0.12)	0.001
TC (mmol/L)						
	HIIT+RT	4.78 (0.48)	4.63 (0.57)	-0.15 (-0.31 to 0.004)	NA	0.469
	HIIT	4.82 (0.78)	4.62 (0.77)	-0.20 (-0.46 to 0.06)	NA	0.369
	HIIT+RT vs HIIT	NA	NA	NA	0.04 (-0.25 to 0.33)	0.002
HDL-C (mmol/L)						
	HIIT+RT	1.39 (0.21)	1.62 (0.19)	0.23 (0.11 to 0.34) ^**^	NA	0.963
	HIIT	1.44 (0.28)	1.60 (0.24)	0.16 (0.03 to 0.28) ^*^	NA	0.608
	HIIT+RT vs HIIT	NA	NA	NA	0.04 (-0.09 to 0.17)	0.011
LDL-C (mmol/L)						
	HIIT+RT	2.51 (0.45)	2.48 (0.32)	-0.04 (-0.22 to 0.15)	NA	0.093
	HIIT	2.60 (0.46)	2.68 (0.64)	0.08 (-0.17 to 0.33)	NA	0.151
	HIIT+RT vs HIIT	NA	NA	NA	-0.15 (-0.43 to 0.13)	0.031
EI (Kcal/day)						
	HIIT+RT	3016 (211)	2987 (182)	-29.68 (-77.55 to 18.18)	NA	0.299
	HIIT	3100 (181)	3066 (187)	-33.89 (-82.53 to 14.74)	NA	0.336
	HIIT+RT vs HIIT	NA	NA	NA	-11.84 (-75.27 to 51.59)	0.004

EI, energy intake; FBG, fasting blood glucose; 2hPG, 2-hour postprandial glucose; HDL-C, high-density lipoprotein cholesterol; LDL-C, low-density lipoprotein cholesterol; TG, triglyceride; TC, total cholesterol; ^*^, *p*<0.05; ^**^, *p*<0.01.

## 4 Discussion

After an 8-week exercise intervention, both the HIIT+RT and HIIT groups showed substantial improvements in body composition, cardiorespiratory fitness and glycolipid metabolism in women with overweight/obesity. Compared to the HIIT group, the combined group demonstrated a superior improvement in muscle mass, VO_2_peak, VC, and VO_2_/HR, but other indicators, although improved, did not show significant differences between groups. Overall, the combined training appeared to offer more benefits to patients with obesity, as indicated by the confidence intervals and effect sizes, but these favorable estimates are accompanied by a certain degree of uncertainty. Therefore, it is important to consider whether the additional benefits of combining HIIT and resistance training are substantial and certain enough to prioritize its promotion among a population of overweight and obesity.

Our research found that both HIIT+RT and HIIT showed significant post-intervention improvements in body composition, including body weight, BMI, body fat, WC, and WHR, which may indicate that both are similar in terms of improvements in body composition. The fat-reducing effect of HIIT may be related to its excess post-exercise oxygen consumption (EPOC), as it significantly increases the total amount and duration of EPOC after exercise, especially during the rapid recovery period and slow recovery period, and this effect helps to continue burning fat after exercise and improves fat loss efficiency (28). Both HIIT and RT can also increase daily energy expenditure by increasing basal metabolic rate, which contributes to long-term weight management and body composition improvement as confirmed by previous studies (29). Additionally, combined training shows more pronounced improvements in muscle mass. This may be because resistance training stimulates muscle growth, leading to increased muscle mass, and increased muscle mass is beneficial for maintaining and increasing metabolic rate, improving body composition, and enhancing physical function (30).

We observed significant changes in cardiorespiratory fitness in patients with obesity in both exercise groups post-intervention, confirming the findings of several previous studies. One meta-analysis showed that HIIT significantly increased the maximum oxygen uptake and decreased RHR in the population with overweight and obesity (31); another study found significant reductions in systolic and diastolic BP following high-intensity exercise (32). However, current research on combined training is limited. From several available evidence, Silva-Reis et al. demonstrated that combined aerobic and resistance training significantly improved lung function in females with overweight and obesity (30) Meanwhile, Zaenker et al. found that HIIT combined with RT significantly improved VO_2_peak in patients with multiple sclerosis (33), and we observed a similar trend in patients with obesity. Overall, the improvements in muscle mass, VO_2_peak, VE, VC, VO_2_/HR, systolic and diastolic BP were more pronounced when RT was combined with the exercise regimen. This suggests that such a combination could be recommended as a new exercise prescription for young people with obesity.

Compared to the HIIT group, the combined training increased VO_2_peak and VC by 1.61 mL/min/kg and 334mL. This may be attributed to the additive effect of HIIT and RT: HIIT can effectively contribute to weight reduction, body fat decrease, and cardiovascular adaptation improvement. On the other hand, RT promotes muscle mass and strength development while enhancing neuromuscular adaptation and improving the heart’s pumping capacity, promoting vascular health. This helps improve blood flow and oxygen delivery efficiency, significantly enhancing participants’ physical performance (34). The combination of these training methods stimulates both the cardiovascular and muscular systems, bringing about more adaptive changes for patients. The cumulative effects of these benefits may lead to more sustained improvements in performance and health benefits, ultimately maximizing participants’ VO_2_peak and VC. A previous meta-analysis indicated that resistance exercise effectively promotes an increase in maximum oxygen uptake (35), and another randomized controlled trial similarly confirmed that aerobic exercise combined with resistance exercise improved respiratory function more than aerobic exercise alone (36). These findings offer a possible explanation for our results. As mentioned, when comparing two training regimens, we should interpret the results cautiously. From the VO_2_peak results, the lower limit of the confidence interval shows a trivial benefit of 0.21 mL/min/kg, while the upper limit exceeds 3.02 mL/min/kg. The lower and upper limits of VC are 91 mL and 576 mL respectively, it also demonstrated gains with some indeterminacy. Overall, our study estimates that incorporating RT into other aerobic training regimens could bring greater improvements in oxygen uptake and respiration capacity for patients with obesity, but the exact extent of additional benefits cannot be accurately estimated from the current data. However, the greater improvements in VO_2_/HR might indicate a valuable synergistic effect.

Post-intervention, both the HIIT+RT and HIIT regimens demonstrated positive impacts on glycemic control and lipid metabolism in subjects with obesity. In terms of blood sugar, both the combined training group and the HIIT group significantly reduced FBG in patients with obesity after the experiment, a result that is also supported by the study of Ahmad et al. (37). Despite the absence of a notable distinction between the groups, the combined training group showed a larger effect size. Additionally, the combined training group significantly reduced 2hPG post-intervention, a benefit not observed in the HIIT group. This finding is novel, as an experiment by Coll-Risco et al. it was noted that a regimen of high-intensity aerobic interval training in conjunction with RT markedly influenced FBG and 2hPG levels in obese rats (38), and we found the same effect in the population of obesity; Differing from another study, Pearson et al. found that Tabata-style high-intensity exercise did not improve FBG and 2hPG in healthy males (39). However, due to the lack of resistance training, smaller sample size, and shorter intervention duration, a direct comparison with the current study is limited. This area requires further, more in-depth research in the future.

On the other hand, Fisher et al.’s study found that 8 weeks of HIIT significantly reduced TG in patients with obesity but did not improve HDL-C (40). Conversely, another study reported that HIIT increased HDL-C without improving other plasma markers (41). Differing from these, both of our exercise groups significantly reduced TG and increased HDL-C in females with obesity post-intervention, a finding also confirmed by a recent randomized controlled trial (42), which showed that HIIT required only 4 weeks to improve dyslipidemia in people with obesity. We did not observe benefits in TC and LDL-C in either group, although there was a decreasing trend. This could be attributed to the insufficient duration of 8 weeks of training to induce significant changes in blood cholesterol levels, as well as variations in the response to exercise among individuals of different ages and levels of obesity, and that some subjects may require a longer period of stimulation for effective improvement in lipid levels, consistent with the findings from Kessler et al.’s study (43). In addition, inadequate dietary control during training may also attenuate the beneficial effects of exercise on TC and LDL-C (44). Future research could involve longer interventions with improved dietary regulation to further investigate the potential health benefits of exercise. A recent study found that time-restricted eating also has a certain impact on treating obesity (22), suggesting that it could be combine with combined training to explore additional benefits.

In summary, compared to aerobic interval training alone, this combined training approach appears to improve the metabolic status of patients with obesity more comprehensively. This could be due to the combined exercise impacting multiple body systems simultaneously, enhancing the function and metabolism of skeletal muscles, promoting fat oxidation and metabolism, improving insulin sensitivity and glucose tolerance, and increasing the body’s antioxidant capacity. These mechanisms interact and collectively promote health, better controlling blood sugar and reducing lipids, thus further decreasing the risk of cardiovascular diseases and other illnesses (45–47).

### 4.1 Strengths and limitations

To the best of our understanding, this research is the inaugural investigation into the impact of HIIT following the Tabata protocol, in conjunction with RT, on women with overweight and obesity issues. Our study’s robustness lies in employing a randomized controlled trial framework for contrasting the effects of two different interventions on the body composition, cardiopulmonary functionality and glycolipid metabolism in women with obesity; secondly, the application of effective and objective measures to assess outcome indicators; furthermore, participants exhibited a high adherence rate throughout the training process and results, with most subjects not reporting significant adverse reactions during or after the exercise period.

Our study also has several limitations. Initially, our study did not assess the prolonged impact of exercise on individuals with obesity, given that the duration of the intervention was limited to only eight weeks. This may not be sufficient to observe the long-term impact of the intervention on some indicators. Another limitation is the recruitment of female participants only, which precludes the analysis of gender differences and limits the applicability of our findings to males and other clinical populations. Additionally, we did not strictly control the diet of the participants, so we cannot rule out the influence of diet on health indicators. The 24-hour dietary recall method may lead to discrepancies between reported and actual food intake due to participants’ inaccurate memory, resulting in potential underestimation or overestimation of energy intake. Moreover, participants’ emotional states, such as stress or anxiety, can also influence their recollection of diet, leading to inaccuracies in reporting. However, during the intervention process, we provided comprehensive professional training and support while utilizing specialized software that offered prompts and standardized images for participants to estimate food portions accurately. This approach effectively minimized reporting errors and ensured precise documentation of their food intake. Prior to and following the intervention, all participants maintained a continuous three-day dietary record estimating and documenting energy intake. The results indicated no significant differences in diet before and after the intervention.

Future research should also investigate the long-term effects and sustainability of combined training interventions, as well as optimize these training methods for different demographic groups, including men and older adults.

## 5 Conclusion

Our study indicates that HIIT combined with RT appears to be a more effective strategy for treating obesity. While both exercise programs significantly improved body composition, cardiorespiratory fitness and glycolipid metabolism in women with overweight/obesity, the combined group had more pronounced benefits than the separate group. This combined training modality seems to elicit better physiological adaptations than HIIT alone. Nonetheless, additional investigations are required to clarify the underlying mechanisms responsible for these particular adaptations.

## Data Availability

The original contributions presented in the study are included in the article/**Supplementary Material**. Further inquiries can be directed to the corresponding author.
