# Supplementary material for: Combined high-intensity interval and resistance training improves cardiorespiratory fitness more than high-intensity interval training in young women with overweight/obesity: a randomized controlled trial
Source: Front Endocrinol (Lausanne). 2024 Nov 11;15:1450944. doi: 10.3389/fendo.2024.1450944 (PMC11586196; doi:10.3389/fendo.2024.1450944)
Supplement: Supplementary file 1 [file Table1.docx]

Supplementary Table S1: Details of the Tabata training program.

| Week | Sessions/week | Exercises | Intensity |
| --- | --- | --- | --- |
| 1-4 weeks | 3 | Jumping jack | 85%-90%HRmax |
|  |  | Crotch clap |  |
|  |  | Knee-to elbow |  |
|  |  | Squat |  |
|  |  | Side Knee raise |  |
|  |  | Kick back |  |
|  |  | Touch foot |  |
|  |  | High knees |  |
| 5-8 weeks | 3 | Jumping jack | 90%-95%HRmax |
|  |  | Crotch clap |  |
|  |  | Cross step |  |
|  |  | Squat |  |
|  |  | Jumping lunge |  |
|  |  | Prone jumping jack |  |
|  |  | Mountain climbers |  |
|  |  | Burpees |  |

Supplementary Table S2: Details of the resistance training program

| Sessions/week | Exercises | Sets | Repetitions | Intensity | Interval | |
| --- | --- | --- | --- | --- | --- | --- |
| 3 | Barbell deadlift  Goblet deep squat  Dumbbell lunge  Machine calf raise | 3 | 10 | 70%1RM | 60s | |
|  | Bench barbell press  Chest press vertical  Dumbbell shoulder press  Dumbbell lateral raises |  |  |  |  |  |
|  | Pull down machine  Seated row  Dumbbell biceps curl  Two-arm rope Extension |  |  |  |  |  |
